# Progressive colloidal clogging mechanism by dendritic build-up in porous media†

**DOI:** 10.1039/d5sm00285k

**Published:** 2025-06-23

**Authors:** Walid Okaybi, Sophie Roman, Cyprien Soulaine

**Affiliations:** a Institut des Sciences de la Terre d'Orléans, ISTO, UMR 7327, Univ Orléans, CNRS, BRGM, OSUC F-45071 Orléans France walid.okaybi@cnrs-orleans.fr walid.okaybi95@gmail.com

## Abstract

Colloidal transport in porous media governs deposition and clogging mechanisms that critically influence flow behavior and impact the efficiency of both natural and industrial systems. However, the role of dendritic structures, a distinct deposition morphology, in this process remains unclear. Understanding the formation and growth of dendrites is essential for advancing clogging dynamics and assessing their impact on permeability. To address this, we perform microfluidic flow experiments and computational fluid analysis to observe and characterize dendrite formation in a heterogeneous tortuous porous domain. Our results reveal a novel clogging mechanism – dendrite clogging – where a single deposition site initiates a structure that extends across the pore space, bridging grains and causing complete clogging. Unlike previously described aggregation-based clogging, which involves multiple deposition sites, dendrite clogging evolves from a single-site deposition. We establish a flow-dependent criterion for dendrite formation by combining hydrodynamic-adhesive torque balance analysis with experimental deposition patterns. Our findings show that dendrites form when front cone stagnation regions are large enough to accommodate multilayer deposition. Moderate flow rates promote dendrite growth, leading to abrupt permeability loss. In contrast, higher flow rates suppress dendrite formation, resulting in a more gradual decline, as captured by the Verma–Pruess permeability–porosity model. Our results provide a predictive model for flow-induced colloidal deposition, with implications for improving filtration systems, groundwater flow, and biomedical microfluidics. Insights into dendrite-driven clogging could lead to methods for reducing clogging in porous systems and optimizing flow performance in diverse applications.

## Abstract 2

Dendritic particle structures form at front stagnation regions and progressively bridge pores, redirecting flow streamlines, which reveals the hydrodynamic impact of clogging in porous microfluidic media.

## Graphical abstract

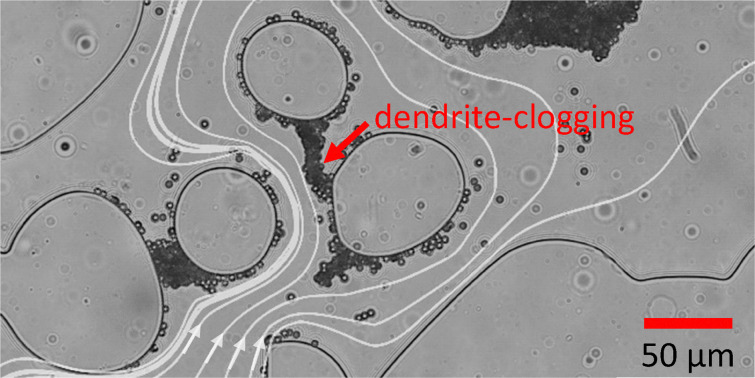

## 1 Introduction

The physics of colloidal transport within confined environments is fundamental to both natural and engineered systems. For example, in groundwater flow, colloid mobility and subsequent pore clogging can influence water quality and alter flow paths within aquifers, directly impacting filtration effectiveness.^1^ Similarly, in geothermal reservoirs, particle clogging reduces the permeability leading to a declined efficiency in performance.^2,3^ This issue also arises in other applications, such as microfiltration membranes used for particle removal,^4^ as well as in micro-engineered systems, porous structures, and micro-reactors, where clogging limits flow and system performance.^5–7^ Therefore, understanding the underlying physics of colloidal deposition and clogging is crucial for overcoming major limitations in various industrial applications.

Particle clogging occurs through three mechanisms:^5^ sieving, where the pore constriction is too small for a particle to flow through;^8–11^ bridging, where multiple particles simultaneously arrive at a constriction and form a bridge;^12–15^ and progressive clogging or aggregation, where particles successively deposit near a wide constriction, narrowing the channel and ultimately causing blockage.^16–19^ The key point to describing the progressive clogging mechanism lies in understanding how particle deposits evolve from initial layers to the formation of multilayer buildups on the walls of the constriction. Achieving effective control over this process, however, presents a significant challenge. It requires a deep understanding of the complex interplay between flow hydrodynamics^20,21^ and adhesion forces, which include electrostatic interactions between particles and surfaces^21,22^ as well as the effects of Brownian motion. The most widely used framework for capturing these electrostatic interactions is the Derjaguin–Landau–Verwey–Overbeek (DLVO) theory.^23,24^ It models adhesion forces as the combined effect of attractive van der Waals forces and repulsive electrostatic interactions from overlapping electric double layers, which dictate particle–particle and particle–surface interactions,^25^ ultimately influencing deposition. Ramachandran and Fogler^26^ investigated the critical conditions for multilayer deposition in a particle–pore surface system, emphasizing the interplay between flow rate and ionic strength. They demonstrated experimentally that higher salt concentrations screen surface charges on flowing sub-micron particles,^27^ weakening the electrostatic repulsion barrier. This reduction allows particles to deposit onto previously deposited ones, which act as additional collectors—solid grains or deposited particles within the porous medium onto which flowing particles can attach due to hydrodynamic and adhesive interactions—leading to multilayer formation. They found that for a particle to deposit, its velocity must exceed a critical threshold to overcome the repulsion barrier and move close enough for adhesion forces to take effect. As ionic strength increases, repulsive forces diminish, lowering the critical velocity required for deposition. At sufficiently high salt concentrations, the repulsion barrier vanishes at small separation distances, meaning that a particle can only deposit if its velocity remains below a certain threshold.

Kusaka *et al.*^28^ studied deposition morphology around a cylindrical obstacle in a microfluidic channel at high salt concentrations, revealing that at low Péclet numbers (low advection relative to diffusion), deposits formed uniformly across the upstream half of the collector. As the Péclet number increases, deposition concentrated at the front stagnation point, eventually forming finger-like dendritic structures. These structures were velocity-dependent: at higher Péclet numbers, dendrites became smaller, and deposition shifted to the rear of the collector. Building on these findings, studies on a one-dimensional (1D) array of aligned pores^29^ and an isolated obstacle^30^ examined the conditions for dendritic growth, emphasizing the effects of salt concentration and flow dynamics. Both studies showed that dendrites form under high salt concentrations and relatively high flow velocities. de Saint Vincent *et al.*^30^ further explained that erosion induces lateral particle detachment, promoting dendritic growth along the upstream centerline of the pore. However, dendritic growth was suppressed as flow velocity increased further due to particle detachment at the centerline. These studies highlight the importance of balancing salt concentration and flow conditions for dendrite formation, with excessively high velocities disrupting their growth. However, they do not define a criterion beyond which dendritic growth is significantly hindered.

Bacchin *et al.*^31^ investigated how different 2D pillar array configurations (straight, connected, and staggered) influence clogging mechanisms. They found that dendritic structures exclusively formed at the entrance of the straight pore array, with no internal clogging, consistent with previous work by Bacchin *et al.*^22^ In contrast, the staggered and connected arrays showed an absence of dendritic build-ups upstream of the pillars. Instead, clogging occurred through successive particle accumulation on the upstream sides of individual pillars, eventually forming a cake layer that blocked the entrance. Despite these insights, the formation of dendrites within heterogeneous porous media and their impact on flow dynamics remain poorly understood. In particular, there is no clear evidence on how dendrite buildup contributes to pore clogging.

Understanding how particle deposition leads to clogging in porous media requires direct visualization of transport mechanisms at the pore scale.^16^ Microfluidic devices offer a powerful tool for this purpose, as they enable the fabrication of transparent two-dimensional (2D)^32^ and three-dimensional (3D)^33^ models with diverse geometric features. These devices allow for high-resolution, real-time observation of transport mechanisms in microchannels while providing rapid analysis, minimal sample consumption, and cost efficiency.^34,35^ While microfluidic devices have been extensively used to study particle deposition and clogging, including in porous-like media with realistic geometries,^6,36,37^ most studies have not addressed the emergence of dendritic structures in such environments or their role in inducing clogging and altering flow behavior. To address this gap, we combine microfluidic experiments with flow simulations to investigate three key aspects of dendrite formation and its impact on clogging in heterogeneous tortuous porous media. First, we examine how dendritic structures progressively develop within the medium, altering flow dynamics and, in some regions, leading to dendrite-induced clogging. Second, we identify the conditions that promote dendrite formation by correlating detailed velocity profiles from flow simulations with the critical velocity for particle detachment, determined through a torque balance between hydrodynamic and adhesive forces. Finally, we assess how dendrite formation and clogging influence permeability loss by analyzing permeability–porosity relationships derived from experimental data.

The structure of the paper is as follows. In Section 2, we describe the materials and methods, including the fabrication of PDMS microfluidic devices, the preparation of particle suspensions, and the setup for flow experiments and imaging. We also present the methodology for determining the critical velocity for particle detachment and the post-processing techniques used in computational fluid flow analysis. In Section 3, we present and discuss our results, starting with experimental evidence of dendrite clogging, followed by an analysis of the conditions that favor dendrite formation, and concluding with an evaluation of its impact on porous media properties. Finally, we summarize the key findings of this study and their broader implications.

## 2 Materials and methods

We present microfluidic systems designed to investigate dendrite formation and its role in clogging within porous media. Our approach includes preparing particle suspensions, setting up experiments with integrated advanced imaging techniques, and identifying critical velocities for particle detachment. We also develop a new methodology to augment our experimental findings using computational microfluidics.

### 2.1 PDMS microfluidic devices

This study employs two microfluidic system designs to explore deposition patterns, focusing on dendritic structures and their influence on pore clogging.

**Fig. 1:**
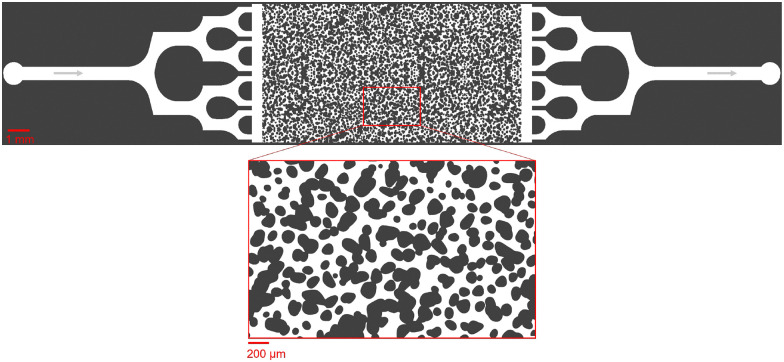
Designed porous domain generated using KLayout, a CAD tool commonly used to create mask layouts for microfabrication processes such as photolithography, showing solid grains in dark gray and pore spaces in white. The porosity *ϕ*_0_ is 0.48, and the permeability *K*_0_ is 2.96 × 10^−12^ m^2^.

The primary design is a porous domain representing a subsurface environment, characterized by pore diameters ranging from 10 to 280 μm, with an average pore width of approximately 100 μm. To construct the porous geometry, we use an image quilting technique,^38^ which randomly samples and stitches patches of grain images to generate a larger, more representative output. This design, illustrated in Fig. 1, features a main channel (0.5 mm wide, 5.5 mm long) that transitions into a diverging network, expanding from two to eight channels to ensure effective flow distribution. To further stabilize the flow before it enters the porous domain, a 6 mm-wide, 0.5 mm-long feeding channel is incorporated. The entire domain has a uniform depth of 20 μm. Spanning 6 mm by 12 mm, the porous domain features a porosity of *ϕ*_0_ = 0.48 and an experimentally determined permeability of *K*_0_ = 2.96 × 10^−12^ m^2^.

The second design is a single-grain collector system, designed to investigate the underlying processes driving the build-up of dendritic structures. The design features a large main channel, 0.5 mm wide and 5 mm long, followed by a 3 mm long converging channel to homogenize the flow. This transitions to a smaller feeding channel, 100 μm wide and 3.6 mm long, with a single grain collector, 20 μm in diameter, positioned at its center. Similar to the porous domain, the single-grain collector system maintains a depth of 20 μm.

We design the geometries using KLayout software and fabricate the master wafer with the predefined geometries through photolithography with SU-8 photoresist.^39^ PDMS is then molded onto the wafer to create replicas of the structures. After curing, we cut the replicas from the mold and punch holes to establish connections between the inlets and outlets. We then treat both the PDMS replica and a glass microscope slide in a plasma chamber for 50 seconds before irreversibly bonding them to assemble the microfluidic device.

### 2.2 Particle suspension

Carboxylate polystyrene latex particles with a diameter of 4.5 μm are purchased from polysciences (2.7% w/v stock solution). The suspension concentration is prepared by diluting the stock solution in a density-matched fluid (78% NaCl solution and 22% glycerine) to minimize the effects of gravity, achieving a final volume fraction of 1.25 × 10^−3^. To control the electrostatic repulsion between particles and between particles and walls, a NaCl concentration of 100 mM is used, which is below the critical coagulation concentration of 157 mM.^40^ Before each experiment, the suspension is sonicated for 30 minutes in an ultrasonic bath to break apart any pre-formed aggregates prior to injection.

### 2.3 Flow experiments and imaging

**Fig. 2:**
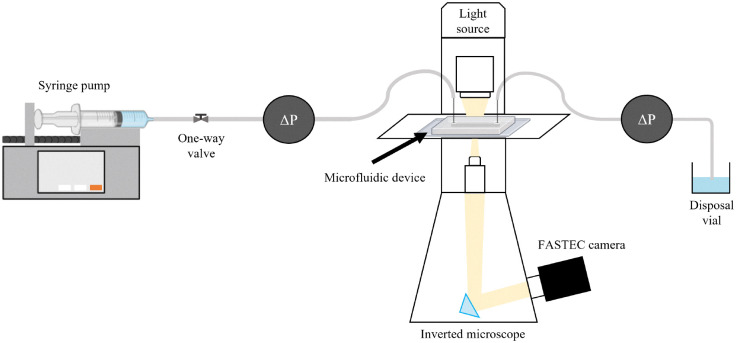
Schematic of the microfluidic setup. A syringe pump drives the particle suspension through the microfluidic device, in which pressure sensors monitor the pressure drop (Δ*P*) across the porous medium. The outlet flow is collected in a vial. An inverted microscope equipped with a high-speed FASTEC camera captures real-time imaging of particle deposition and clogging dynamics. Some elements were created with BioRender.com.

We inject particle suspension into the microfluidic device previously saturated with 100 mM NaCl solution using a KD Scientific syringe pump at a controlled flow rate, as shown in Fig. 2. All the experimental parameters with their corresponding values are listed in Table 1. The flow conditions are carefully controlled to ensure a creeping flow regime, with a Reynolds number Re = *ρVL*/*μ* < 1. *V* represents the characteristic velocity at the feeding channel just before the single-grain collector and the porous domain. *L* is the characteristic length chosen as the depth (*h*) across both microfluidic devices. Additionally, we evaluate the Péclet number (Pe = (3π*d*_p_*μLV*)/(*k*_B_*T*)), which quantifies the contribution of advection to diffusion for suspended particles. In our experiments, the Péclet number exceeds 2.9 × 10^5^, indicating that particle transport is dominated by advection rather than diffusion. We also evaluate the Stokes number (St = (*ρ*_p_*d*_p_^2^*V*)/(18*Lμ*)), which quantifies the influence of inertial effects on particle transport. It is defined as the ratio of the particle's characteristic time scale to the fluid's characteristic time scale and is calculated to be St < 6.5 × 10^−3^. Since this value is well below 10^−2^, inertial effects are negligible for the suspended particles.^41^

Parameters for fluid properties, particle characteristics, surface properties, and force calculations in the experimental and simulation setup. The annotations p and s refer to the particle and surface collector, respectively

**Table:** 

Fixed parameters	Value
Particle and carrier fluid density, *ρ*	1050 kg m^−3^
Depth of the microfluidic device, *h*	20 μm
Dynamic viscosity, *μ*,	1.83 mPa s
Particle diameter, *d*_p_	4.5 μm
Boltzmann constant, *k*_B_	1.38 × 10^−23^ J K^−1^
Temperature, *T*	293 K
Adhesive force (particle–surface), *F*^p–s^_A_	2.3 × 10^−8^ N
Adhesive force (particle–particle), *F*^p–p^_A_	3.4 × 10^−8^ N
Poisson ratio (surface), *υ*_s_	0.5
Poisson ratio (particle), *υ*_p_	0.35
Young modulus (surface), *E*_s_	2 MPa
Young modulus (particle), *E*_p_	3 GPa
Elastic constant (surface),^43^ 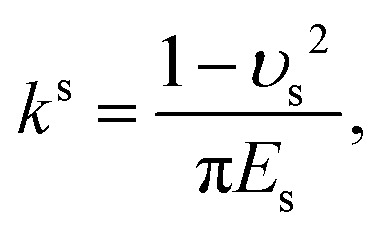	1.2 × 10^−7^
Elastic constant (particle),^43^ 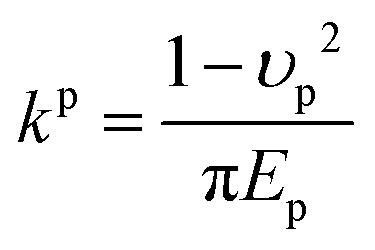	9.3 × 10^−11^
Particle–surface Young's modulus,^43^ 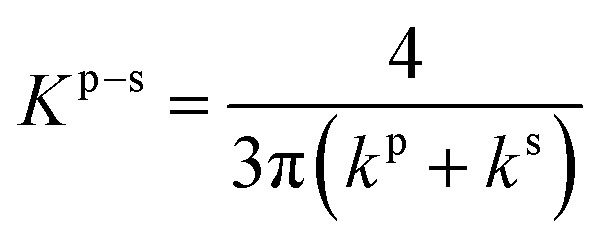	3.56 MPa
Particle–particle Young's modulus,^43^ 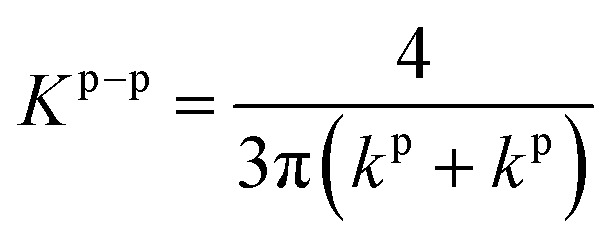	2.28 GPa

**Table:** 

Variable parameters	Value
Flow rate through porous domain, *Q*	8, 20 μL min^−1^
Flow rate through single-grain collector, *Q*	1, 5, 10 μL min^−1^

The microfluidic device is positioned on an inverted microscope (Nikon ECLIPSE Ti2 microscope platform) connected to a high-speed FASTEC (HS7) camera to capture image sequences at a specific depth using magnifications of 2×, 4×, 10×, and 20× after a certain number of pore volumes is injected. At selected pore volumes during the experiment, we recorded sequences of 300 images at 300 frames per second. These images were then averaged to filter out moving particles and highlight the deposited particles only. To reproduce a two-dimensional image of the entire porous domain, we use an image stitching technique.^42^ Porosity over time, *ϕ*(*t*), is measured by adaptive binarization of these images, which allows us to track changes in porosity due to particle deposition. Since porosity is estimated from 2D images, our analysis does not account for the full 3D stacking of particles along the channel depth (20 μm). Given the particle diameter (4.5 μm), up to approximately 4 to 5 particles can stack vertically. In regions with partial stacking (*e.g.*, two layers), this assumption overestimates the solid volume by around 55%, leading to a porosity underestimation of approximately 15%. However, in regions where particle stacking spans the full channel height, the 2D assumption becomes valid, and the porosity inaccuracy is negligible. Moreover, optical observations reveal clear redirection of local streamlines due to clogging, suggesting that particle deposits often span the entire depth of the channel. This implies that, in most cases, the porosity estimation remains only minimally affected in our study.

Additionally, we have installed two inline pressure sensors from FLUIGENT on both sides of the microfluidic device to monitor pressure differentials continuously throughout the experiment. These pressure readings are instrumental in calculating the permeability of the porous domain over time, *K*(*t*), which is then normalized against the initial permeability, *K*_0_.

### 2.4 Critical velocity for particle–surface and particle–particle detachment

We aim to identify a critical velocity that determines whether a particle will attach to or detach from a collector surface or an already deposited particle. Establishing this criterion helps us understand the mechanisms driving different deposition patterns and the processes underlying their formation. As aggregates grow in linear low-shear regimes, we consider the stability of particles at stagnation regions along with the flow direction. Given that rolling is the predominant mechanism of particle detachment,^21,44,45^ the torque on an attached particle results from a balance between adhesive forces acting normal to the surface (or deposited particle) and drag forces acting tangentially. The adhesive force tends to bind a particle to the surface, while the drag force tends to mobilize it. Under laminar conditions, the drag force acting on a spherical particle reads as *F*_D_ = (1.7)(3π*μd*_p_*V*^f^_*x*_), where *V*^f^_*x*_ is the fluid velocity.^46,47^ The drag force is effective at 1.4(*d*_p_/2), reading an applied torque of *T*_D_ = 1.4(*d*_p_/2)*F*_D_.^48^ On the other hand, the adhesive force, *F*_A_, is calculated as the sum of van der Waals and electric double-layer potentials (see Section S1 of the ESI†) acting between particles or between a particle and a surface, divided by an approximated separation distance of 0.2 nm.^49^ The adhesive torque for attached particles is expressed as *T*_A_ = *F*_A_·*l*_*x*_, where *l*_*x*_ represents the contact radius between the particle and either a surface or another particle. The contact radius is defined as *l*_*x*_ = (*F*_A_(*d*_p_/2)/4*K*^p–i^)^1/3^ and depends on the particle size and the material properties of both the particle and the surface.^21^ Here, *K*^p–i^ represents the composite Young's modulus, which describes the elastic properties at the contact interface—either between two particles or between a particle and the collector surface.^43^ The critical detachment velocity, (*V*^c^_*x*_), is obtained by balancing the hydrodynamic and the adhesive torques. It reads:1
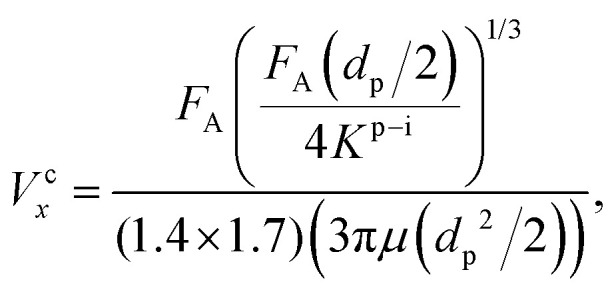
where all the parameters with their corresponding values are defined and listed in Table 1. The subscripts p and i denote the particle and either another particle or the surface, respectively.

### 2.5 Post-processing using computational microfluidics

We perform direct fluid flow simulations to complement and enhance our experiments by providing deeper insights into the fluid dynamics within our microfluidic devices. These simulations generate detailed velocity profiles, helping to identify stagnation regions that favor particle deposition. Additionally, they enable us to map the redirection of fluid streamlines after accounting for experimentally observed particle deposition and clogging. To achieve this, we first convert an experimental binary image of the entire porous domain, with a resolution of 5 μm pixel^−1^, into a computational grid. In this grid, permeability is penalized by assigning very low values to grains and deposited particles, while high values are assigned to pore spaces, following the approach described by Soulaine *et al*.^50^ and Soulaine and Tchelepi.^51^ Fluid flow is then simulated using porousMedia4Foam, an OpenFOAM-based package dedicated to multi-scale flow and transport in porous media.^52^ We solve the 2D Stokes equation while incorporating the Hele–Shaw correction term, which accounts for the effect of the microchannels finite depth. The Hele–Shaw approximation simplifies the flow into a depth-averaged 2D equation by introducing an effective permeability term proportional to *h*^2^/12, where *h* is the microchannel's depth. This correction ensures that the simulated flow regime remains consistent with the experimental conditions. The simulations assume saturated, incompressible flow with no-slip boundary conditions at grain surfaces. Fixed inlet velocities, along with constant fluid density and viscosity, are set to match experimental parameters presented in Table 1.

## 3 Results and discussion

In this section, we demonstrate how the continuous growth of dendrites leads to dendrite-induced clogging. We then analyze the conditions that favor dendrite formation using both a single-grain collector and a tortuous porous domain at different flow rates. Finally, we investigate the impact of flow rate on the hydraulic properties of the porous domain.

### 3.1 Experimental evidence of dendrite clogging

We introduce the particle suspension with a 100 mM NaCl concentration into the porous domain at two flow rates: 8 μL min^−1^ and 20 μL min^−1^. These flow rates correspond to velocities of 1.1 mm s^−1^ and 2.7 mm s^−1^, respectively, in the feeding channel just before the porous domain. The high salt concentration in our flow experiments reduces electrostatic repulsion between particles, promoting multi-layer particle deposition.^27^ The chosen flow rates correspond to a Péclet number range of 𝒪(10^5^–10^6^) within the porous domain. According to Bacchin *et al.*,^22^ this range is conducive to multi-layer particle build-up in stagnation regions, where low shear allows for the formation of dendritic structures. In our porous domain—characterized by highly connected, tortuous pathways and varying grain sizes—these conditions could similarly facilitate dendritic growth. A key question arises: can these structures develop sufficiently to bridge neighboring grains and ultimately clog the pores? To analyze the resulting clogging mechanisms, we capture time-sequenced images of the experiments, allowing us to investigate how different flow rates influence pore clogging and deposition patterns.

**Fig. 3:**
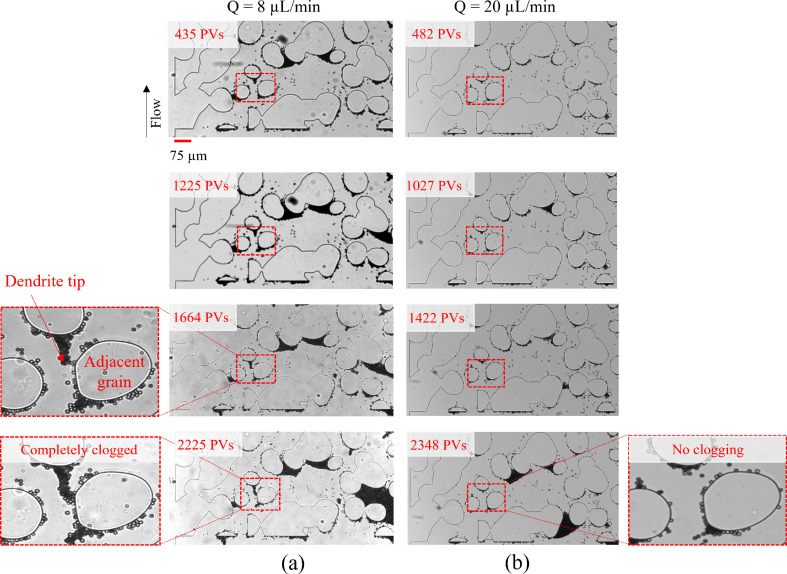
Averaged images from experiments conducted at two flow rates, (a) 8 μL min^−1^ and (b) 20 μL min^−1^, capturing a section of the porous domain at different pore volumes and highlighting distinct clogging mechanisms. In (a), multiple clogging regions emerge, with a particular focus on the progressive development of dendritic clogging. An initial dendrite forms after 435 PVs (highlighted in a red dotted box), extending upstream by 1225 PVs. By 1664 PVs, the dendrite tip advances further toward the adjacent grain (annotated in the magnified side image), eventually leading to complete pore clogging as the structure continues to grow, constricting the local flow pathway. In (b), multiple clogging events occur, but no dendrite formation or dendrite-induced clogging is observed in the same region, even after injecting 2348 PVs.

**Fig. 4:**
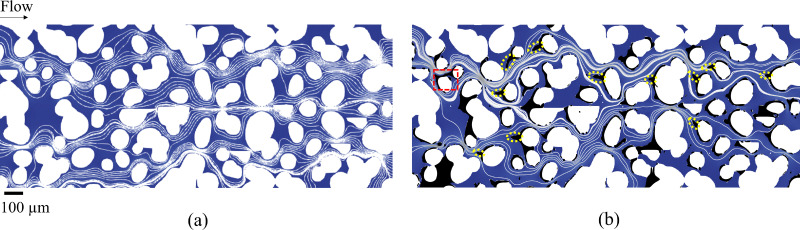
Streamlines extracted from fluid flow simulations at an 8 μL min^−1^ flow rate, showing particle deposits in black and grains in white. (a) Flow field at the beginning of the experiment, illustrating the initial streamline configuration. (b) Flow field after 2225 PVs of particle suspension injection, where dendritic structures (circled in yellow) are seen growing into the pore space. These deposits progressively distort the surrounding streamlines. The red-dotted box highlights a region where dendrite-induced clogging causes a complete redirection of particle flow, contrasting with the initial pattern in (a). Additional clogging mechanisms throughout the domain are also observed to contribute to local flow redirection.

We identify a new progressive clogging mechanism termed “dendrite clogging”. Unlike typical clogging patterns involving progressive aggregation and deposition at two deposition sites at the entrance of the pore,^16,17^ dendrite clogging arises from the presence of dendrites (elongated structures) that grow across the pore from a single deposition site—the tip of the dendrite. To illustrate this process, Fig. 3a shows averaged images from a sequence of captured frames, highlighting a specific region of interest within the porous domain. These images correspond to different pore volumes injected (PVs) of the particle suspension at a flow rate of 8 μL min^−1^. After 435 PVs, multiple clogging events appear in the region, with a notable initial dendrite forming at the front stagnation zone of a grain facing the flow (highlighted by a dotted red box). By 1225 PVs, additional particles accumulate on the existing deposits, causing the dendrite to extend upstream into the pore. After 1664 PVs, we observe a further extension of the dendrite significantly reducing the gap between its tip and the adjacent grain (both annotated in the figure) without complete pore-clogging, allowing particles to still flow through, as shown in Movie_Figure_3a_8_μL_min_1664_PVs in the ESI.† Finally, by 2225 PVs, the continuous buildup of the dendritic structure leads to complete pore-clogging and redirection of the local pathway, which is shown in Movie_Figure_3a_8_μL_min_2225_PVs in the ESI.† In addition, we have observed and tracked the progression of dendrite-induced clogging across several regions of the porous domain (see Section S2 of the ESI†). Agbangla *et al.*^29^ observed that the orientation of dendrites is based on the flow direction in a 2D straight array of pores. To validate this in a more complex and tortuous porous domain, we extract streamlines from velocity profiles obtained from numerical simulations at an 8 μL min^−1^ flow rate (Fig. 4), before and after injecting 2225 PVs of the particle suspension, with deposited particles shown in black. Fig. 4a shows the initial flow field at the beginning of the experiment. Fig. 4b illustrates the flow field after clogging has occurred. Our results confirm that dendrite orientation, highlighted in the yellow-circled areas, closely follows the streamlines shaped by the flow direction and gradually modifies them as the dendrites grow. As highlighted in the red-dotted box, the continuous build-up of dendrites leads to clogging, forcing a complete redirection of the local flow path of particles, which strongly contrasts with the initial streamline configuration observed in Fig. 4a.

In the same region of interest within the porous domain (Fig. 3b), the experiment conducted at a flow rate of 20 μL min^−1^ exhibits multiple clogging events but no dendrite formation or dendrite-induced clogging. From 482 PVs to 2348 PVs, the absence of a dendritic structure within the highlighted red box suggests that dendrite clogging does not occur at this higher flow rate. This can be attributed to the increased shear at 20 μL min^−1^, which is 2.5 times higher than at 8 μL min^−1^, effectively limiting dendrite growth. This finding aligns with previous experimental observations,^28,30^ where a higher Péclet number was shown to inhibit the development of elongated dendritic structures along the centerline of the collector.

### 3.2 Conditions for dendrite formation

In the previous section, we demonstrated how dendrite formation progressively leads to clogging as more particles accumulate with increasing PVs. We also observed that higher flow rates generate greater shear stress at front stagnation regions, disrupting particle–particle attachment and preventing dendritic growth. To further investigate the conditions that promote dendrite formation, we focus on a single-grain collector geometry, which allows us to isolate and quantify the factors governing dendrite emergence in porous media. We emphasize that the single-grain collector was not designed to replicate the full complexity of the porous medium, but rather to isolate the hydrodynamic and adhesive conditions necessary to initiate dendritic build-up. Although the 20 μm cylindrical grain is smaller than the grains in the porous domain, this controlled geometry enables a focused analysis of the initial stages, promoting dendrite formation and multilayer growth.

**Fig. 5:**
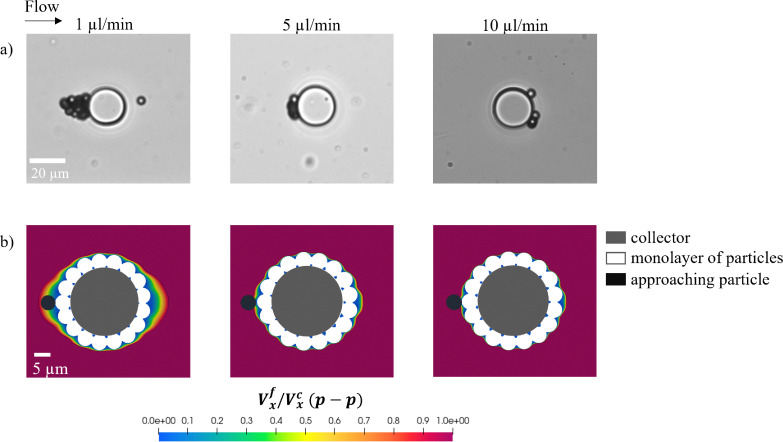
(a) Images captured after 2500 PVs under 100 mM salt concentration, illustrating the different deposition patterns achieved on a single-grain collector geometry at flow rates ranging from 1 μL min^−1^ to 10 μL min^−1^. At 1 μL min^−1^, particles progressively build up at the upstream stagnation region of the collector surface, forming a dendrite. At 5 μL min^−1^, a monolayer of depositions forms with no particle–particle attachment observed. At 10 μL min^−1^, no deposits are observed at the upstream stagnation region; instead, particles decelerate in this region, roll along the collector surface, and attach at the rear stagnation region. (b) Simulated fluid velocity fields normalized by the critical velocity for particle–particle detachment (*V*^f^_*x*_/*V*^c^_*x*_(p–p)). The simulations include both the collector and an explicit monolayer of deposited particles. At 1 μL min^−1^, the upstream stagnation region is wide enough to accommodate an additional particle, promoting multilayer buildup. As the flow rate increases, this region compresses and becomes too narrow to support further deposition, explaining the absence of dendrites at higher flow rates.

To achieve this, we conduct experiments by injecting particle suspensions at flow rates ranging from 1 μL min^−1^ to 10 μL min^−1^, corresponding to velocities between 8.3 mm s^−1^ and 83 mm s^−1^ in the feeding channel just before the single-grain collector. We maintain the same salt concentration (100 mM) as in the porous domain experiments to ensure consistency. To analyze deposition patterns, we record image sequences after 2500 PVs injected through the single-grain collector geometry, roughly aligning with the endpoint of the porous domain experiments for comparison. In Fig. 5a, at a flow rate of 1 μL min^−1^, particles predominantly attach at the front stagnation region, facing the flow, leading to a progressive buildup and the formation of dendrites. At 5 μL min^−1^, deposition is limited to a monolayer on the collector surface in the upstream stagnation region, with no significant particle–particle attachment. At 10 μL min^−1^, no deposition occurs at the upstream stagnation region. Instead, as particles approach this zone, they experience a velocity drop, causing them to roll along the collector's sides before settling at the rear stagnation region.

To establish a quantitative basis for our experimental observations, we calculate the critical detachment velocity using eqn (2), which balances the adhesive torque that binds a particle to a collector surface or previously deposited particle against the drag torque that mobilizes it. Our results suggest that for a particle to attach to a collector surface, its velocity (*V*^f^_*x*_) must remain below the threshold *V*^c^_*x*_(p–s) = 8.3 mm s^−1^. In comparison, attachment to a previously deposited particle requires a lower threshold, with *V*^f^_*x*_ needing to stay below *V*^c^_*x*_(p–p) = 1.6 mm s^−1^. Both values are calculated at a separation distance of one particle radius. In Table 2, we present the normalization of the simulated fluid flow velocity (*V*^f^_*x*_) with the calculated critical velocities *V*^c^_*x*_(p–s) and *V*^c^_*x*_(p–p). Our results show that particle–collector surface attachment is favored in the three experiments performed as *V*^f^_*x*_/*V*^c^_*x*_(p–s) < 1. However, the location of these attachments is highly dependent on the hydrodynamic force. At flow rates of 1 μL min^−1^ and 5 μL min^−1^, particles tend to deposit at the front stagnation regions where *V*^f^_*x*_/*V*^c^_*x*_(p–s) remains relatively low. In contrast, at 10 μL min^−1^, when the fluid velocity approaches this threshold (*V*^f^_*x*_/*V*^c^_*x*_(p–s) ≈ 1), particles begin to roll along the surface, reducing their contact area and weakening adhesive forces. This rolling motion continues until they reach the rear stagnation point, where adhesive forces are re-established, promoting stable deposition. Particle deposition in the rear stagnation zones of the collector observed in our experiments aligns with the findings of Kusaka *et al.*,^28^ which indicate that at sufficiently high flow rates, particles preferentially accumulate at the rear of the collector.

**Table 2:** Ratios of simulated fluid velocity (*V*^f^_*x*_) to the critical detachment velocity for particle–collector surface attachment (*V*^c^_*x*_(p–s)) and particle–particle attachment (*V*^c^_*x*_(p–p)) at a separation distance of one particle radius, across flow rates ranging from 1 μL min^−1^ to 10 μL min^−1^

Flow rate (μL min^−1^)	1	5	10
*V* ^f^_*x*_/*V*^c^_*x*_(p–s)	0.08	0.4	0.8
*V* ^f^_*x*_/*V*^c^_*x*_(p–p)	0.4	2	4

For dendritic structures to develop, a monolayer of deposits must first form at the front stagnation regions, observed only at 1 μL min^−1^ and 5 μL min^−1^. However, dendrite formation occurred only at the lower flow rate (1 μL min^−1^), where the fluid velocity remains below the threshold for particle–particle attachment, where *V*^f^_*x*_/*V*^c^_*x*_(p–p) < 1 (Table 2). To analyze particle–particle attachment, we assume in our analysis that an initial monolayer of particles forms along the collector surface, represented as “monolayer of particles” in Fig. 5b. This monolayer serves as a prerequisite for initiating multilayer deposition by providing a foundation for further buildup.

In Fig. 5b, we perform fluid flow simulations to extract the velocity profile and normalize it by the critical velocity threshold for particle–particle detachment. This allows us to define cone-shaped stagnation regions that promote multilayer build-up. In these regions, adhesive forces dominate over hydrodynamic forces, promoting particle aggregation and the formation of multilayer deposits, which eventually can develop into elongated dendritic structures. In contrast, higher shear forces outside these zones suppress aggregation, limiting particle–particle attachment along the sides of the grain. We also highlight the effect of increasing flow rate on the compression of these front stagnation regions. At 1 μL min^−1^, the stagnation region is large enough to accommodate one particle diameter, illustrated by the “approaching particle” shown in Fig. 5b. However, as the flow rate increases, these regions compress, becoming too small to accommodate even a single particle. Since dendrites formed only at 1 μL min^−1^, as experimentally observed in Fig. 5a, this suggests that dendrite formation requires a stagnation region large enough to accommodate at least one particle diameter, promoting directional multilayer build-up—a flow-dependent criterion that highlights the critical role of the cone-shaped stagnation zone. Our findings align with those of van der Wee *et al.*,^53^ who reported that active microrollers preferentially accumulate in hydrodynamically defined low-velocity zones around cylindrical obstacles, analogous to the cone-shaped stagnation regions identified in our study. While their system involves active particles, it reinforces the broader conclusion that flow-defined structures govern preferential particle accumulation regardless of the deposition mechanism.

**Fig. 6:**
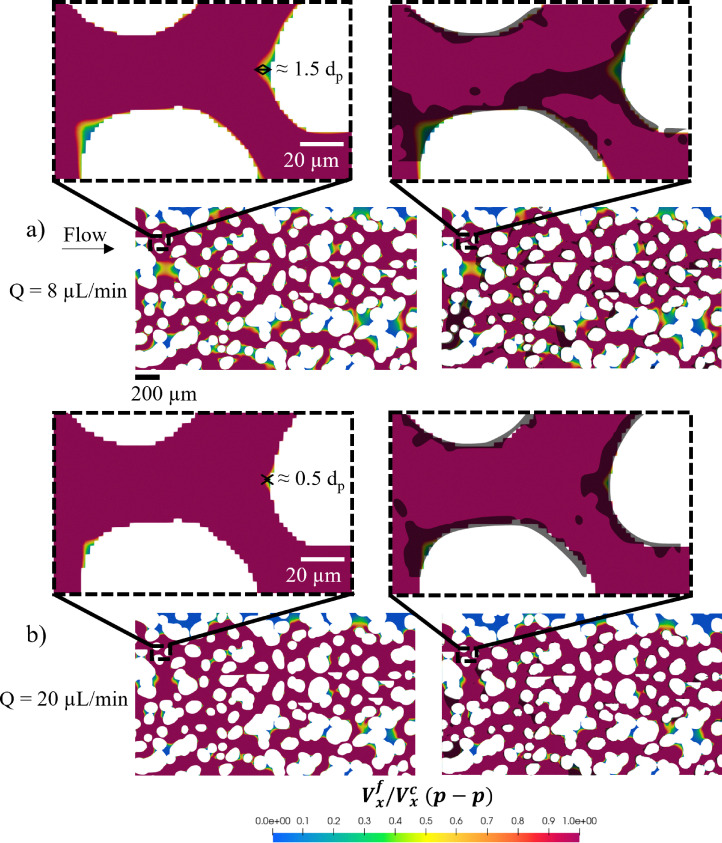
Fluid flow velocity profiles obtained by simulations and normalized by the critical velocity for particle–particle detachment (*V*^f^_*x*_/*V*^c^_*x*_(p–p)) at flow rates of 8 μL min^−1^ (a) and 20 μL min^−1^ (b). (a) The magnified image shows a stagnation region that accommodates approximately 1.5 particle diameters. The adjacent image overlays the experimental deposition pattern onto the normalized velocity profile, highlighting dendrite formation and build-up in the front stagnation regions. The progression of dendrite growth leading to clogging is shown in the magnified image. (b) The magnified image shows a stagnation region that accommodates only 0.5 particle diameters. The adjacent image shows the superimposition of the experimental deposition pattern on the normalized velocity profile, illustrating the absence of dendrites in the front stagnation regions within the porous domain. This emphasizes the critical role of stagnation region size in initiating dendrite growth, as the region is too small to accommodate a single particle diameter.

In the context of the porous domain, we similarly assume that an initial monolayer of particles forms along the grain surfaces, serving as a base for further multilayer buildup. Applying this analysis—and the flow-dependent criterion extracted from the single-grain collector geometry—to the porous domain, we find that at 8 μL min^−1^ the cone-shaped stagnation region, annotated in the magnified image in Fig. 6a, can accommodate approximately 1.5 particle diameters. If this criterion indeed holds in the porous geometry, dendrite formation should occur, which is confirmed by superimposing the final particle deposits from the experiment onto the *V*^f^_*x*_/*V*^c^_*x*_(p–p) profile. In contrast, at 20 μL min^−1^, as shown in the magnified image in Fig. 6b, the front stagnation region compresses to the point where it can no longer accommodate even a single particle. Consistently, superimposing the final particle deposits onto the *V*^f^_*x*_/*V*^c^_*x*_(p–p) profile reveals no dendritic structures but rather a predominant monolayer of particles surrounding the grains.

From both the single-grain collector and porous domain analyses, we conclude that dendrite formation requires a cone-shaped stagnation region large enough to accommodate at least one particle diameter, promoting directional multilayer build-up.

### 3.3 Impact on porous media properties

We have shown that dendrites form under specific conditions and play a role in clogging. Understanding how particle deposition and clogging affect porosity and permeability is essential. Porosity is determined from binary images of the porous domain captured at different pore volumes of particles injected (PVs), while permeability changes are inferred from pressure drop measurements (Δ*P*) taken during experiments performed at flow rates of 8 μL min^−1^ and 20 μL min^−1^.

**Fig. 7:**
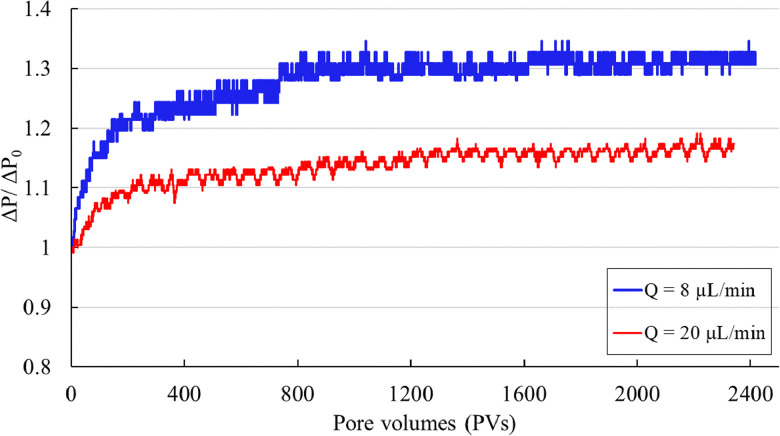
The normalized pressure drop (Δ*P*/Δ*P*_0_) across the porous domain after injecting multiple pore volumes (PVs) is shown for two flow rates, *Q* = 8 μL min^−1^ and *Q* = 20 μL min^−1^. In both experiments, Δ*P*/Δ*P*_0_ builds up due to particle deposition and clogging, with consistently higher values at *Q* = 8 μL min^−1^. During the first 800 PVs, the pressure difference rises rapidly before gradually stabilizing between 1400 PVs and 2400 PVs in both cases.

In Fig. 7, the normalized pressure difference (Δ*P*/Δ*P*_0_) increases over time in both experiments, reflecting progressive particle deposition and clogging. At a flow rate of 8 μL min^−1^, the normalized pressure difference is consistently higher than at 20 μL min^−1^, indicating greater deposition and clogging at lower flow rates due to reduced particle velocity. Both experiments show a rapid increase in Δ*P*/Δ*P*_0_ during the first 800 PVs, followed by a gradual rise until it stabilizes between 1400 PVs and 2400 PVs. The experiment is terminated after 2400 PVs, as further changes in Δ*P* become negligible.

We compare our experimental data to established permeability models to quantify the relationship between permeability and porosity. The Kozeny–Carman equation is a widely used model that relates permeability to porosity based on an idealized flow representation through packed granular media.^54,55^ However, it assumes uniform particle deposition and a homogeneous pore structure, which does not accurately capture the complexity of clogging in our system. In particular, our experiments reveal that permeability decline is influenced by localized dendritic growth and heterogeneous deposition patterns, leading to deviations from the smooth permeability reduction predicted by the Kozeny–Carman model.

Given these limitations, we use the Verma–Pruess model, a power law extension of the Kozeny–Carman model, originally proposed by Verma and Pruess^56^ and later reformulated by Ott *et al.*^57^ This model was initially developed to describe permeability reduction caused by mineral precipitation and biofilm accumulation, where solid deposits gradually obstruct pore spaces.^58^ Since both mineral precipitation and dendrite formation and clogging follow the same localized, structure-dependent pattern of modifying and blocking the flow, the Verma–Pruess model is a good choice for describing permeability loss in our system. This nonlinear model better reflects the permeability–porosity relationship observed in our experiments:2
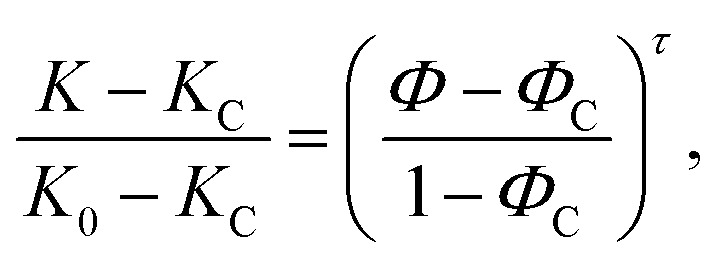
where *Φ* = *ϕ*/*ϕ*_0_ represents the normalized porosity, where *ϕ* is the actual porosity and *ϕ*_0_ is the initial porosity. Likewise, *K* is the actual permeability, *K*_0_ is the initial permeability, and *K*_C_ is the critical (or minimum) permeability reached due to particle deposition and clogging at a critical reduced porosity, *Φ*_C_.

**Fig. 8:**
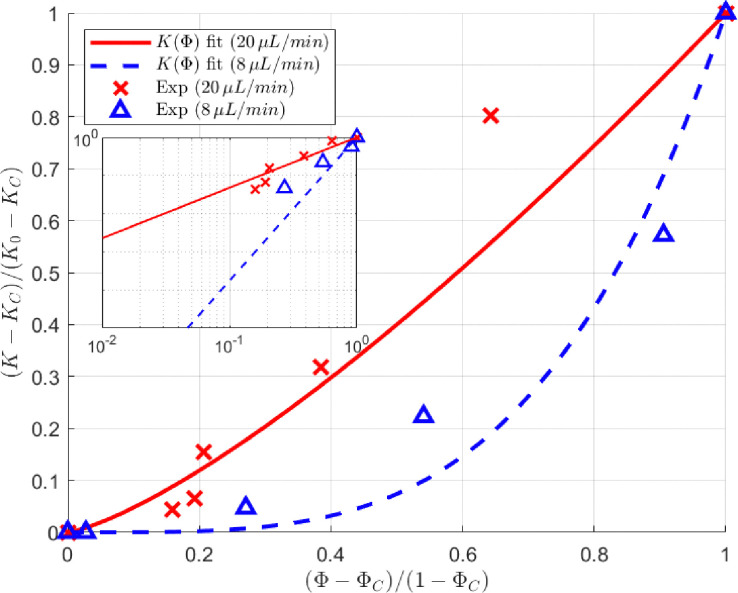
The permeability–porosity relationships (*K*(*Φ*)) describing the experimental observations for the two flow rates *Q* = 8 μL min^−1^ (blue) and *Q* = 20 μL min^−1^ (red) are shown along with their corresponding fitting curves. These curves accurately capture the experimental trends using the Nonlinear Least Squares method with a robust bisquare weighting, achieving *R*-squared values of 0.995 and 0.982 for *Q* = 8 μL min^−1^ and *Q* = 20 μL min^−1^, respectively. The data for *Q* = 8 μL min^−1^ clearly shows a steeper permeability decline, with an exponent *τ* = 3.75, compared to *τ* = 1.32 for *Q* = 20 μL min^−1^. This indicates a more pronounced particle deposition and clogging effect at lower flow rates. An inset shows the same data on a log–log scale, confirming the power-law behavior through the linearity of the trend in logarithmic coordinates.

A key advantage of the Verma–Pruess model is that it incorporates *K*_C_ and *Φ*_C_, which allow us to describe the experimentally observed stabilization of permeability and porosity. By assigning *Φ*_C_ = 0.864 and *Φ*_C_ = 0.944, corresponding to the final porosities observed at the end of the 8 μL min^−1^ and 20 μL min^−1^ experiments, we obtain *K*_C_/*K*_0_ values of 0.764 and 0.867, respectively. Fig. 8 presents the experimentally obtained permeability–porosity relationship for the two flow rates, 8 μL min^−1^ and 20 μL min^−1^, and their respective fitting curves. Each data point corresponds to a distinct pore volume injected during a single continuous experiment at a given flow rate. Separate experiments were conducted for each flow rate, and the data were analyzed independently. In the final plot, we selected data points that correspond to similar injected pore volumes across experiments to facilitate meaningful comparison between flow rates. The fitting curves effectively describe the experimental data trends, yielding exponents of *τ* = 3.75 ± 0.66 for 8 μL min^−1^ and *τ* = 1.32 ± 0.27 for 20 μL min^−1^. The higher exponent at 8 μL min^−1^ reflects a more abrupt permeability loss, consistent with more pronounced effects of particle depositions and clogging inferred from the pressure measurements. To confirm the power-law behavior, a log–log representation of the same data is provided as an inset in Fig. 8, and demonstrating that the experimental relationship is not linear on a standard plot.

These findings highlight the critical role of flow rate in governing clogging mechanisms and deposition patterns. At a moderate flow rate of 8 μL min^−1^, dendritic structures emerge in the front stagnation regions begin to alter local streamlines and redirect flow even before complete pore blockage occurs, as shown in Fig. 4 and the Video (Movie_Figure_3a_8 μL_min_1664_PVs, ESI†). In some regions, these dendrites continue to grow, as observed in Fig. S3 and the ESI,† ultimately leading to full pore blockage and significant redirection of the local flow due to dendrite-induced clogging. These effects—both before and after dendrite clogging—combined with the fact that lower particle velocities at 8 μL min^−1^ promote increased deposition through additional clogging mechanisms, contribute to a more pronounced permeability decline. In contrast, at the higher flow rate of 20 μL min^−1^, dendrite formation is suppressed, and particle deposition is less pronounced due to higher velocities, resulting in a more gradual permeability decline. Together, these results demonstrate the effectiveness of the Verma–Pruess model in capturing deposition patterns and underscore how hydrodynamic conditions regulate particle accumulation and clogging behavior in porous media.

## 4 Conclusions

From industrial uses like microfluidic filtration to natural processes like groundwater filtration, clogging in porous media plays a critical role in many diverse systems. Our study provides new insights into how dendritic structures drive clogging in porous media, highlighting a distinct mechanism where deposition extends progressively from a single deposition site to bridge pore spaces. Unlike clogging by aggregation, dendrite clogging emerges due to flow-dependent stagnation zones that enable multilayer deposition within them. This finding advances our understanding of permeability loss in complex porous environments and suggests that clogging dynamics are not solely dictated by particle accumulation but also by the evolving interplay between hydrodynamics and deposition patterns.

Despite these advances, key questions remain. The impact of particle surface properties, such as charge and roughness, on dendrite stability is not yet fully understood. Another open question concerns the role of fixed-pressure conditions in dendrite formation. Under such conditions, the flow rate gradually decreases as clogging progresses. Assuming the system initially satisfies our flow-dependent criterion for dendritic growth, we expect that the velocity weakening over time would reduce the adjacent high-shear zones responsible for sustaining directional growth. This reduction in shear could promote multilayer deposition along the sides of the growing dendrite, eventually burying the tip and transitioning the structure into a flattened or radially symmetric deposit. Additionally, while our experiments provide direct observations in two-dimensional porous networks, a natural extension would be to investigate the extent to which dendrite formation and dendrite-driven clogging translate to three-dimensional environments. Addressing these aspects will refine predictive models and inform strategies for mitigating clogging in complex porous systems. Extending these insights to broader porous environments could enhance filtration efficiency, optimize subsurface flow, and improve microfluidic performance, ultimately contributing to better clogging control in both natural and engineered systems.

## Author contributions

Walid Okaybi: writing – original draft, visualization, validation, methodology, investigation, formal analysis, conceptualization. Sophie Roman: writing – review and editing, validation, methodology, supervision, conceptualization. Cyprien Soulaine: writing – review and editing, validation, supervision, project administration, methodology, software, funding acquisition, conceptualization.

## Conflicts of interest

There are no conflicts to declare.

## Supplementary Material

SM-021-D5SM00285K-s001

SM-021-D5SM00285K-s002

SM-021-D5SM00285K-s003

## Data Availability

Data for this article, including processed experimental images and the parameters used to establish the porosity–permeability relationship, are available in “Dataset for Progressive colloidal clogging mechanism by dendritic build-up in porous media” at https://doi.org/10.5281/zenodo.15044323.

## References

[cit1] Ryan J. N., Elimelech M. (1996). Colloids Surf., A.

[cit2] You Z., Bedrikovetsky P., Badalyan A., Hand M. (2015). Geophys. Res. Lett..

[cit3] Maya Fogouang L., André L., Soulaine C. (2025). J. Comput. Phys..

[cit4] Gopal R., Kaur S., Ma Z., Chan C., Ramakrishna S., Matsuura T. (2006). J. Membr. Sci..

[cit5] Dressaire E., Sauret A. (2017). Soft Matter.

[cit6] Liu Q., Zhao B., Santamarina J. C. (2019). J. Geophys. Res.: Solid Earth.

[cit7] Chen Y., Sabio J. C., Hartman R. L. (2015). J. Flow Chem..

[cit8] Sauret A., Barney E. C., Perro A., Villermaux E., Stone H. A., Dressaire E. (2014). Appl. Phys. Lett..

[cit9] Sauret A., Somszor K., Villermaux E., Dressaire E. (2018). Phys. Rev. Fluids.

[cit10] Duchêne C., Filipe V., Huille S., Lindner A. (2020). Soft Matter.

[cit11] Majekodunmi O. T., Hashmi S. M. (2022). Sci. Rep..

[cit12] Marin A., Lhuissier H., Rossi M., Kähler C. J. (2018). Phys. Rev. E.

[cit13] Vani N., Escudier S., Sauret A. (2022). Soft Matter.

[cit14] Hafez A., Liu Q., Finkbeiner T., Alouhali R. A., Moellendick T. E., Santamarina J. C. (2021). Sci. Rep..

[cit15] Vani N., Escudier S., Jeong D.-H., Sauret A. (2024). Phys. Rev. Res..

[cit16] Wyss H. M., Blair D. L., Morris J. F., Stone H. A., Weitz D. A. (2006). Phys. Rev. E: Stat., Nonlinear, Soft Matter Phys..

[cit17] Dersoir B., de Saint Vincent M. R., Abkarian M., Tabuteau H. (2015). Microfluid. Nanofluid..

[cit18] Dersoir B., Schofield A., de Saint Vincent M. R., Tabuteau H. (2019). J. Membr. Sci..

[cit19] Dersoir B., Schofield A. B., Tabuteau H. (2017). Soft Matter.

[cit20] Bacchin P., Aimar P., Sanchez V. (1995). AIChE J..

[cit21] Torkzaban S., Bradford S. A., Walker S. L. (2007). Langmuir.

[cit22] Bacchin P., Marty A., Duru P., Meireles M., Aimar P. (2011). Adv. Colloid Interface Sci..

[cit23] Derjaguin B. V. (1941). Acta Physicochim. URSS.

[cit24] Verwey E. J. W. (1947). J. Phys. Chem..

[cit25] Maya Fogouang L., André L., Leroy P., Soulaine C. (2024). J. Comput. Phys..

[cit26] Ramachandran V., Fogler H. S. (1998). Langmuir.

[cit27] Ryde N., Kallay N., Matijević E. (1991). J. Chem. Soc., Faraday Trans..

[cit28] Kusaka Y., Duval J. F., Adachi Y. (2010). Environ. Sci. Technol..

[cit29] Agbangla G. C., Climent É., Bacchin P. (2012). Sep. Purif. Technol..

[cit30] de Saint Vincent M. R., Abkarian M., Tabuteau H. (2016). Soft Matter.

[cit31] Bacchin P., Derekx Q., Veyret D., Glucina K., Moulin P. (2014). Microfluid. Nanofluid..

[cit32] Duffy D. C., McDonald J. C., Schueller O. J., Whitesides G. M. (1998). Anal. Chem..

[cit33] Accardo A., Courson R., Riesco R., Raimbault V., Malaquin L. (2018). Addit. Manuf..

[cit34] Manz A., Harrison D. J., Verpoorte E. M., Fettinger J. C., Paulus A., Lüdi H., Widmer H. M. (1992). J. Chromatogr. A.

[cit35] Whitesides G. M. (2006). Nature.

[cit36] Soumane Y. , PhD thesis, Université Grenoble Alpes [2020-…], 2021

[cit37] He H., Xiong X., Wu T., Hu R., Chen Y.-F., Yang Z. (2025). Sep. Purif. Technol..

[cit38] Cheng L. , *[SigGraph2002] Image Quilting/Texture Synthesize*, https://www.mathworks.com/matlabcentral/fileexchange/35828-siggraph2002-image-quilting-texture-synthesize, 2024, MATLAB Central File Exchange. Retrieved November 7, 2024

[cit39] McDonald J. C., Duffy D. C., Anderson J. R., Chiu D. T., Wu H., Schueller O. J., Whitesides G. M. (2000). Electrophoresis.

[cit40] Guo Y., Tang N., Lu L., Li N., Hu T., Guo J., Zhang J., Zeng Z., Liang J. (2024). Chemosphere.

[cit41] Espinosa-Gayosso A., Ghisalberti M., Ivey G. N., Jones N. L. (2012). J. Fluid Mech..

[cit42] Preibisch S., Saalfeld S., Tomancak P. (2009). Bioinformatics.

[cit43] Johnson K. L., Kendall K., Roberts A. (1971). Proc. R. Soc. London, Ser. A.

[cit44] Bergendahl J., Grasso D. (1998). Colloids Surf., A.

[cit45] Tsai C.-J., Pui D. Y., Liu B. Y. (1991). J. Aerosol Sci..

[cit46] Goldman A. J., Cox R. G., Brenner H. (1967). Chem. Eng. Sci..

[cit47] O'neill M. (1968). Chem. Eng. Sci..

[cit48] Sharma M. M., Chamoun H., Sarma D. S. R., Schechter R. S. (1992). J. Colloid Interface Sci..

[cit49] Israelachvili J. N. , Intermolecular and surface forces, Academic Press, 2011

[cit50] Soulaine C., Tchelepi H. A. (2016). Transp. Porous Media.

[cit51] Soulaine C., Maes J., Roman S. (2021). Front. Water.

[cit52] Soulaine C., Pavuluri S., Claret F., Tournassat C. (2021). Environ. Modell. Software.

[cit53] van der Wee E. B., Blackwell B. C., Balboa Usabiaga F., Sokolov A., Katz I. T., Delmotte B., Driscoll M. M. (2023). Sci. Adv..

[cit54] Kozeny J. (1927). Sitzungsber. Akad. Wiss. Wien, Math.-Naturwiss. Kl., Abt. 2B.

[cit55] Carman P. (1939). J. Agric. Sci..

[cit56] Verma A., Pruess K. (1988). J. Geophys. Res.: Solid Earth.

[cit57] Ott H., Roels S., De Kloe K. (2015). Int. J. Greenhouse Gas Control.

[cit58] Hommel J., Coltman E., Class H. (2018). Transp. Porous Media.

