# Supplementary material for: Progressive colloidal clogging mechanism by dendritic build-up in porous media
Source: Soft Matter. 2025 Jun 23;21(28):5687–98. doi: 10.1039/d5sm00285k (PMC12369462; doi:10.1039/d5sm00285k)
Supplement: SM-021-D5SM00285K-s003 [file SM-021-D5SM00285K-s003.pdf]

Supplementary Information (SI) for Soft Matter.
This journal is © The Royal Society of Chemistry 2025

Supplementary Materials
Progressive colloidal clogging mechanism by dendritic build-up in porous media

Walid Okaybi[1*], Sophie Roman[1], Cyprien Soulaine[1]

[1]Institut des Sciences de la Terre d'Orléans, ISTO, UMR 7327, Univ Orléans, CNRS, BRGM, OSUC, F-45071 Orléans, France
[*]To whom correspondence should be addressed: walid.okaybi@cnrs-orleans.fr

1 DLVO interaction potential approximations

We apply DLVO theory to approximate particle-particle and particle-surface interactions. It represents the sum of attractive potentials due to van der Waals forces and repulsion potentials due to the interaction of the electric double layers:

$$V^{DLVO} = V^{VdW} + V^{EDL}.$$

In Equation 1 we present the particle-particle interaction potential for particles of radius a_p separated by a distance h (where $h \ll a_p$), as proposed by Schenkel and Kitchener [7]. Additionally, Equation 2 presents the particle-surface interaction potential as described by Hogg et al. [4]:

$$V_{pp}^{DLVO} = -\frac{Aa_p}{12h} + \frac{\epsilon_0\epsilon_r a_p \phi_p^2}{2}\left(\ln\left(1+\exp(-\kappa h)\right)\right), \tag{1}$$

$$V_{ps}^{DLVO} = -\frac{Aa_p}{6h}\left(\frac{1}{1+14h/\lambda}\right) + \frac{\epsilon_0\epsilon_r a_p}{4}(\phi_p^2+\phi_s^2)\left(\frac{2\phi_p\phi_s}{\phi_p^2+\phi_s^2}\ln\left(\frac{1+e^{-\kappa h}}{1-e^{-\kappa h}}\right) + \ln\left(1-\exp(-2\kappa h)\right)\right), \tag{2}$$

where all the parameters with their corresponding values are defined and listed in Table 1. Additionally, we plot the DLVO potentials for particle-particle and particle-surface interactions as a function of the separation distance (Fig. 1).

2 Dendrite clogging at multiple locations within the porous domain

Fig. 2 presents averaged experimental images capturing the progressive formation of a dendritic clog in a specific region of the porous domain at a flow rate of 8 µL/min, as the number of

Table 1: Parameters and their corresponding values used in the DLVO interaction potential approximations. The annotations p, m, and s refer to the particle, medium, and PDMS surface.

**Fixed parameters**	**Value**
Particle radius, a_p	2.25 µm
Vacuum permittivity, ε_0	8.85×10^{-12} C^2/Jm
	78.5 for water [1]
Medium dielectric constant, ε_r	40.2 for glycerol [1]
	70.1 (weighted average)
Boltzmann constant, k_B	1.38×10^{-23} J/K
Temperature, T	293 K
Avogadro's number, N_A	6.023×10^{23}
Electron charge, e	1.6×10^{-19} C
	$A_{polystyrene} = 6.5 \times 10^{-20}$ J [3]
	$A_{PDMS-surface} = 4.4 \times 10^{-20}$ J [2]
Hamaker constant, A	$A_{water} = 3.7 \times 10^{-20}$ J [3]
	$A_{glycerol} = 1.38 \times 10^{-20}$ J [5]
$A_{33} = A_{medium}$	3.19×10^{-20} J (weighted average)
$A_{131} = (\sqrt{A_{33}} - \sqrt{A_{11}})(\sqrt{A_{33}} - \sqrt{A_{11}})$ [5]	2.7×10^{-21} J for polystyrene-polystyrene interaction
$A_{132} = (\sqrt{A_{33}} - \sqrt{A_{11}})(\sqrt{A_{33}} - \sqrt{A_{22}})$ [5]	7.3×10^{-21} J for polystyrene-surface interaction
Retardation length, λ	100×10^{-9} m
Solution Ionic strength, I	100 mM
Measured particles zeta potential, ϕ_p	-21.3 mV
PDMS surface zeta potential, ϕ_s	-23 mV [6]
Reciprocal Debye length, $\kappa = \left(\frac{2\times10^3 N_A e^2 I}{\varepsilon_0 \varepsilon_r K_B T}\right)^{\frac{1}{2}}$	1.2×10^9 m

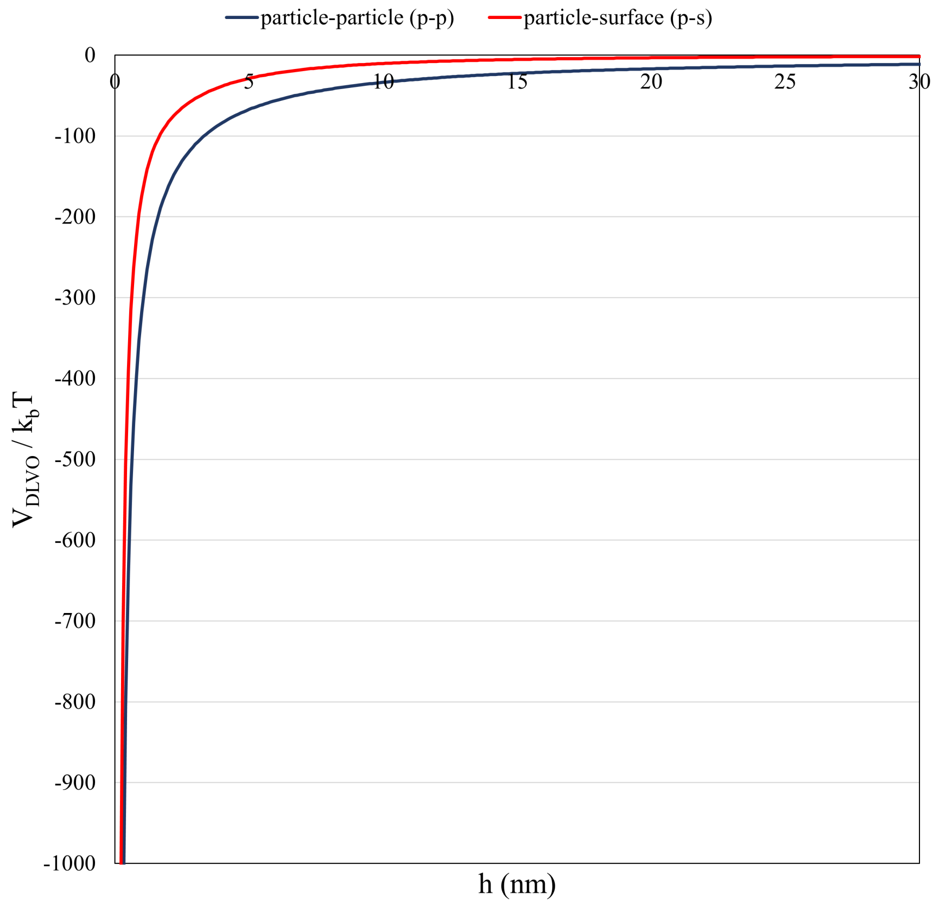

Figure 1: DLVO profiles for particle-particle and particle-surface interactions as a function of separation distance (h) in nanometers (nm).

injected pore volumes (PVs) increases, leading to the complete clogging of the local flow pathway. Fig. 3 further illustrates dendritic clogging occurring in two distinct regions: (a) after 2370 PVs and (b) after 2261 PVs, emphasizing the variability in dendrite-clogging dynamics within the porous domain.

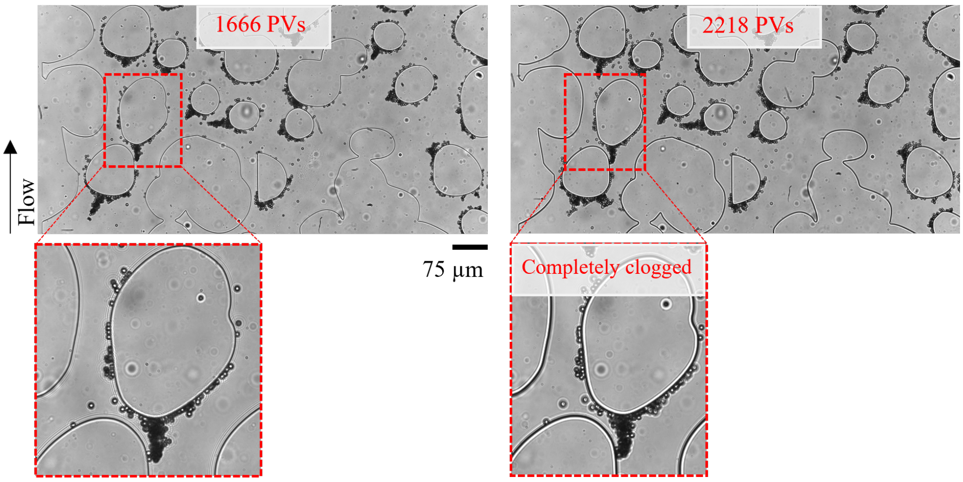

Figure 2: Averaged experimental images at a flow rate of 8 μL/min, showing the progressive formation of a dendritic clog in a specific region of the porous domain over successive pore volumes of particle injection (PVs).

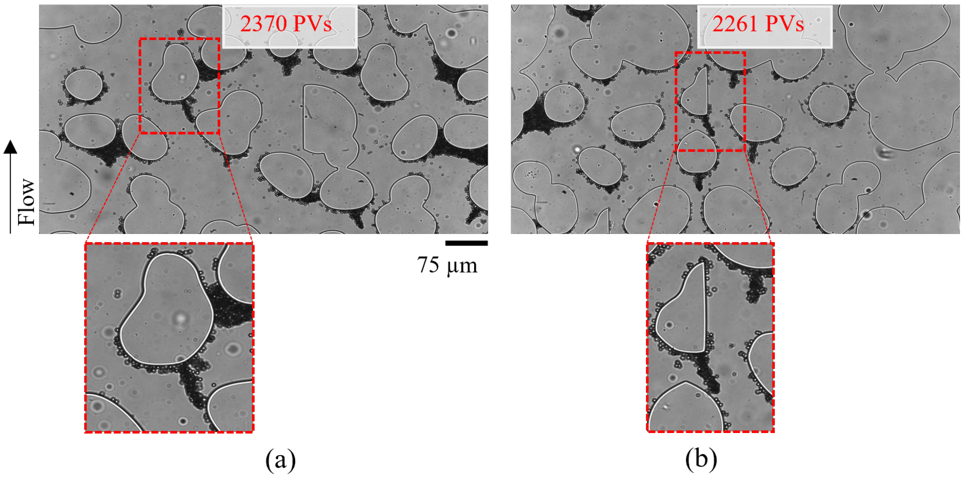

Figure 3: Averaged experimental images at a flow rate of 8 μL/min, illustrating dendritic clogging in two distinct regions of the porous domain: (a) after 2370 pore volumes (PVs) of particle injection and (b) after 2261 PVs.

3 Movies of the experiments

The movies were recorded using a FASTEC camera at 271 fps and are presented at a playback speed of 10 frames per second:

- Movie corresponding to Figure 3(a) in the main manuscript after 1664 PVs injected: `Movie_Figure_3a_8uL_min_1664_PVs`.

- Movie corresponding to Figure 3(a) in the main manuscript after 2225 PVs injected: `Movie_Figure_3a_8uL_min_2225_PVs`.

References

[1] Gosta Akerlof. Dielectric constants of some organic solvent-water mixtures at various temperatures. *Journal of the American Chemical Society*, 54(11):4125–4139, 1932.

[2] Calum J Drummond and Derek YC Chan. van der waals interaction, surface free energies, and contact angles: dispersive polymers and liquids. *Langmuir*, 13(14):3890–3895, 1997.

[3] Yong-Kuan Gong, Kenichi Nakashima, and Renliang Xu. A novel method to determine effective charge of polystyrene latex particles in aqueous dispersion. *Langmuir*, 17(9): 2889–2892, 2001.

[4] RTWDW Hogg, To Wo Healy, and Douglas W Fuerstenau. Mutual coagulation of colloidal dispersions. *Transactions of the Faraday society*, 62:1638–1651, 1966.

[5] Jacob N Israelachvili. *Intermolecular and surface forces*. Academic press, 2011.

[6] B Mustin and B Stoeber. Single layer deposition of polystyrene particles onto planar polydimethylsiloxane substrates. *Langmuir*, 32(1):88–101, 2016.

[7] JH Schenkel and JA Kitchener. A test of the derjaguin-verwey-overbeek theory with a colloidal suspension. *Transactions of the Faraday Society*, 56:161–173, 1960.
